# Characteristics and predictors of mortality on haemodialysis in Brazil: a cohort of 5,081 incident patients

**DOI:** 10.1186/s12882-022-02705-x

**Published:** 2022-02-23

**Authors:** Ana Beatriz Lesqueves Barra, Ana Paula Roque-da-Silva, Maria Eugenia F. Canziani, Jocemir R. Lugon, Jorge Paulo Strogoff-de-Matos

**Affiliations:** 1 Postgraduation Program in Medical Sciences, Universidade Federal Fluminense, Niterói, Rio de Janeiro, Brazil; 2 Fresenius Medical Care Brazil, Rio de Janeiro, Brazil; 3 Escola Paulista de Medicina, Universidade Federal de São Paulo, São Paulo, Brazil; 4 Nephrology Division, Department of Medicine, Universidade Federal Fluminense, Av. Marquês do Paraná 303, 2 andar, Niterói, Rio de Janeiro, Zip Code 24033-900 Brazil

**Keywords:** End-stage renal disease, Haemodialysis, Epidemiology, Survival analysis, Brazil

## Abstract

**Background:**

Although Brazil has one of the largest populations on haemodialysis (HD) in the world, data regarding patients’ characteristics and the variables associated with risk of death are scanty.

**Methods:**

This is a retrospective analysis of all adult patients who initiated on maintenance HD at 23 dialysis centres in Brazil between 2012 and 2017. Patients were censored after 60 months of follow-up or at the end of 2019.

**Results:**

A total of 5,081 patients were included in the analysis. The median age was 59 years, 59.4% were men, 37.5% had diabetes as the cause of kidney failure. Almost 70% had a central venous catheter (CVC) as the initial vascular access, about 60% started dialysis in the hospital, and fluid overload (FO) by bioimpedance assessment was seen in 45% of patients. The 60-month survival rate was 51.4%. In the Cox regression analysis, being older (*P*<0.0001), starting dialysis in the hospital (*P*=0.016), having diabetes as the cause of kidney failure (*P*=0.001), high alkaline phosphatase (*P*=0.005), CVC as first vascular access (*P*=0.023), and FO (*P*<0.0001) were associated with higher death risk, whereas higher body mass index (*P*=0.015), haemoglobin (*P*=0.004), transferrin saturation (*P*=0.002), and serum albumin (*P*<0.0001) were associated with better survival. The same variables, except initial CVC use (*P*=0.14), were associated with death risk in an analysis of subdistribution proportional hazards ratio including the competing outcomes.

**Conclusions:**

The present study gives an overview of a large HD population in a developing country and identifies the main predictors of mortality, including some potentially modifiable ones, such as unplanned initiation of dialysis in the hospital and fluid overload.

**Supplementary Information:**

The online version contains supplementary material available at 10.1186/s12882-022-02705-x.

## Introduction

The prevalence of kidney failure is increasing worldwide. Haemodialysis (HD) is the most common treatment method for this population. Brazil has one of the largest populations on HD in the world, and the number of patients has increased from nearly 87,000 to 133,000 over the last 10 years [1].

The life expectancy of patients undergoing HD remains dramatically shorter than in the general population, although a trend for improvement in the survival over the years has been recently documented in the USRDS [2] and the ERA-EDTA Registry [3]. According to 2020 USRDS Annual Report, 58% of the patients on HD are alive after 3 years and only 41% after 5 years of therapy [2]. Of note, the mortality rate is substantially high in the first year after dialysis initiation, decreases markedly in the second year, and exhibits a steady increase over time [2, 4]. In Brazil, mortality data of the patients on HD are limited since the nationwide registry is a sample that comes from voluntary reports by the clinics. The latest reported data show an annual gross mortality rate of 19.5% [1].

Several factors have been associated with mortality of HD patients, including older age, presence of diabetes and cardiovascular disease, and some potentially modifiable factors such as the presence of a central venous catheter, hypervolemia, and anaemia [5, 6]. Moreover, differences in patient characteristics, environment factors and health care plans can potentially impact the outcome [7]. Overall, the knowledge about HD patients and factors influencing mortality come mainly from developed countries. This information, which is scarce in Brazil, could be a useful reference for further studies and healthcare policy. Thus, the present study aimed to evaluate the main predictors of mortality in a large HD population of a developing country.

## Methods

This is a retrospective analysis of all incident HD patients from July 01, 2012 to June 30, 2017 in 23 Fresenius Medical Care dialysis centres in Brazil. The dialysis centres were distributed among 6 out of 27 states of Brazil (Rio de Janeiro, São Paulo, Minas Gerais, Bahia, Pernambuco, and Brasília - Federal District). All data were extracted from the European Clinical Dialysis Database (EuCliD®), a standardized electronic database used by the participating centres.

Patients under 18 years of age and those who underwent peritoneal dialysis or kidney transplantation before starting HD were excluded. Demographic, clinical and laboratory data on the admission to the clinic were used to define the baseline characteristics of the patients. At the onset of the maintenance haemodialysis, the patients had the body composition analysed by bioimpedance spectroscopy (BIS) - BCM®, Fresenius Medical Care. They were classified as fluid overloaded if pre-dialysis excessive extracellular volume was higher than 13% for women and 15% for men [8].

Data were censored at 60 months of follow-up or on December 31, 2019. Data were also censored in cases of transfer to another dialysis centre, kidney transplant, migrating to peritoneal dialysis, recovery of renal function or abandonment of therapy. The primary outcome was all-cause mortality. This study was performed in accordance with the Declaration of Helsinki and was approved by the local ethics committee under the number CAAE 76623317.1.0000.5243. No written informed consent was requested due to the retrospective nature of the study.

### Statistical analysis

Kolmogorov-Smirnov test was used to test for the distribution of variables. Continuous variables with normal distribution were expressed as mean ± standard deviation or as median and interquartile range otherwise. Categorical variables were presented as frequencies. Comparisons between means were made using the unpaired t test or Mann-Whitney test and comparisons between frequencies were made using the chi-square test. The survival rate was calculated by Kaplan-Meier method and curves were compared by the Log-Rank test. The hazard ratios for death were estimated by Cox proportional hazards regression. Initially, univariate analysis was performed for each variable of interest and, subsequently, only those variables that showed *p*-values lower than 0.10 in the univariate assessment were included in the multivariate analysis. Additionally, similar analyses were done with adjustment for competing outcomes (kidney transplantation, migration to peritoneal dialysis, and kidney function recovery) using the subdistribution proportional hazards model described by Fine and Gray [9]. *P*-values < 0.05 were considered statistically significant. All analyses were performed using SPSS version 18.0 for Windows (IBM©, Chicago, IL, USA), except the subdistribution hazard ratio analysis by the Fine-Gray method, which was performed using the freely available software R version 4.0.2.

## Results

A total of 5,081 incident patients on HD in the period were included in this analysis. The baseline characteristics of the patients are in Table 1. The median age was 59 years and 59.4% were men. Diabetes, followed by hypertension, was the most common cause of kidney failure. Almost 70% of patients had a central venous catheter as the initial vascular access, and nearly 60% initiated dialysis in the hospital. More than half of the patients had the treatment funded by the public healthcare system. Patients who had the treatment funded by private insurance were older and with a higher prevalence of diabetes. Comparisons between demographic and clinical characteristics of patients with public and private healthcare insurances are shown in in the Supplementary Appendix (Table S1).

**Table 1:** Baseline characteristics of patients (*n*= 5,081)

Gender male, n (%)	3,018 (59.4)
Age, years	59 (47 – 69)
Non-white race/ethnicity, n (%)	2,877 (56.6)
Primary cause of kidney failure, n (%)	
Diabetes	1,906 (37.5)
Hypertension	1,317 (25.9)
Glomerulonephritis	547 (10.8)
Polycystic kidney disease	193 (3.8)
Others	357 (7.0)
Unknown	761 (15.0)
Public healthcare insurance, n (%)	2,951 (58.1)
Early referral to nephrologist, n (%)	1,982 (39.0)
Place of first dialysis session	
Hospital	3,060 (60.2)
Dialysis centre	1,321 (26.0)
No information	700 (13.8)
Initial vascular access, n (%)	
Native arteriovenous fistula	1,509 (29.7)
Graft	35 (0.7)
Temporary catheter	2,847 (56.0)
Tunnelled catheter	690 (13.6)
Hepatitis B infection, n (%)	33 (0.6)
Hepatitis C infection, n (%)	136 (2.7)
HIV infection, n (%)	48 (0.9)
Erythropoietin use, n (%)	2,231 (43.9)
Haemoglobin, g/dL	9.8 (8.4 – 11.3)
Transferrin saturation, %	24 (17 – 34)
Ferritin, ng/mL	362 (155 – 722)
BUN, mg/dL	54 (43 – 69)
Serum albumin, g/L	36 (33 – 40)
Potassium, mEq/L	5.0 (4.5 – 5.7)
Phosphorus, mg/dL	4.6 (3.7 – 5.7)
Corrected calcium, mg/L	9.0 (8.6 – 9.5)
Intact parathyroid hormone, pg/mL	262 (125 – 509)
Alkaline phosphatase, UI/L	96 (73 – 136)
Pre-HD Systolic BP, mmHg	142 (129 – 156)
Pre-HD Diastolic BP, mmHg	79 (70 – 85)
Body mass index, Kg/m^2^	23.7 (21.0 – 27.1)
Bioimpedance spectroscopy assessment	
Lean mass, %	49.3 (39.8 – 60.4)
Fat mass, %	34.0 (25.7 – 41.4)
Excessive extracellular volume, %	12.9 (4.8 – 21.1)
Fluid overload, n (%)	1,721 (45.4)

Values are expressed as frequency (%) or median (interquartile range). *BUN* blood urea nitrogen; *HD* haemodialysis; *BP* blood pressure

**Fig. 1:**
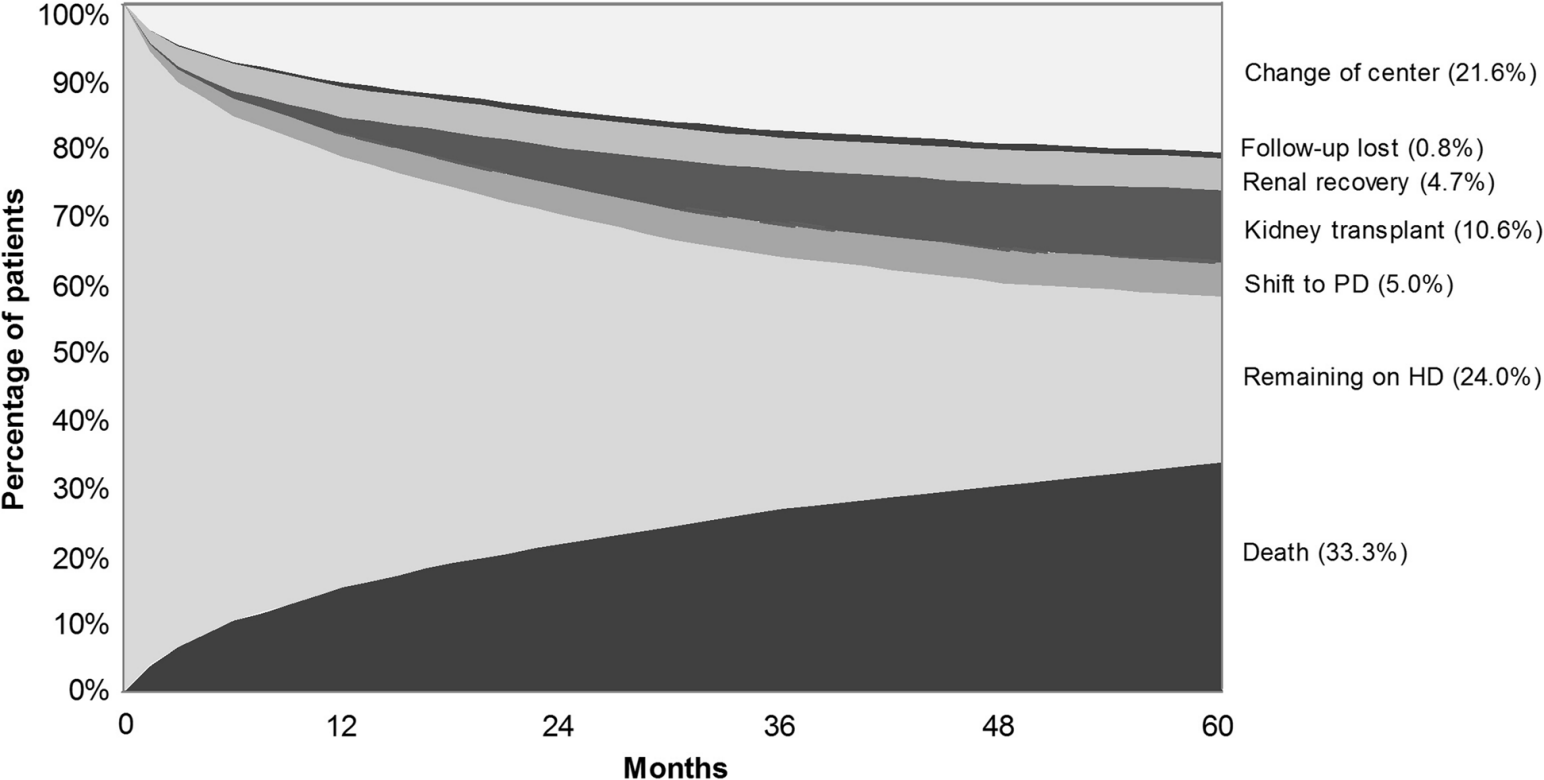
Percentage of patients remaining on haemodialysis and the cumulative frequency of exit from the therapy, according to the cause, along the 60-month period of follow-up

By the end of the 60-month period, 33.3% of the patients followed had died, 24.0% remained on HD, 21.6% were transferred to other dialysis units, 10.6% had received a kidney transplant, 5.0% had migrated to peritoneal dialysis, 4.7% recovered from renal failure, and 0.8% had their follow-up lost, as shown in Fig. 1.

**Fig. 2:**
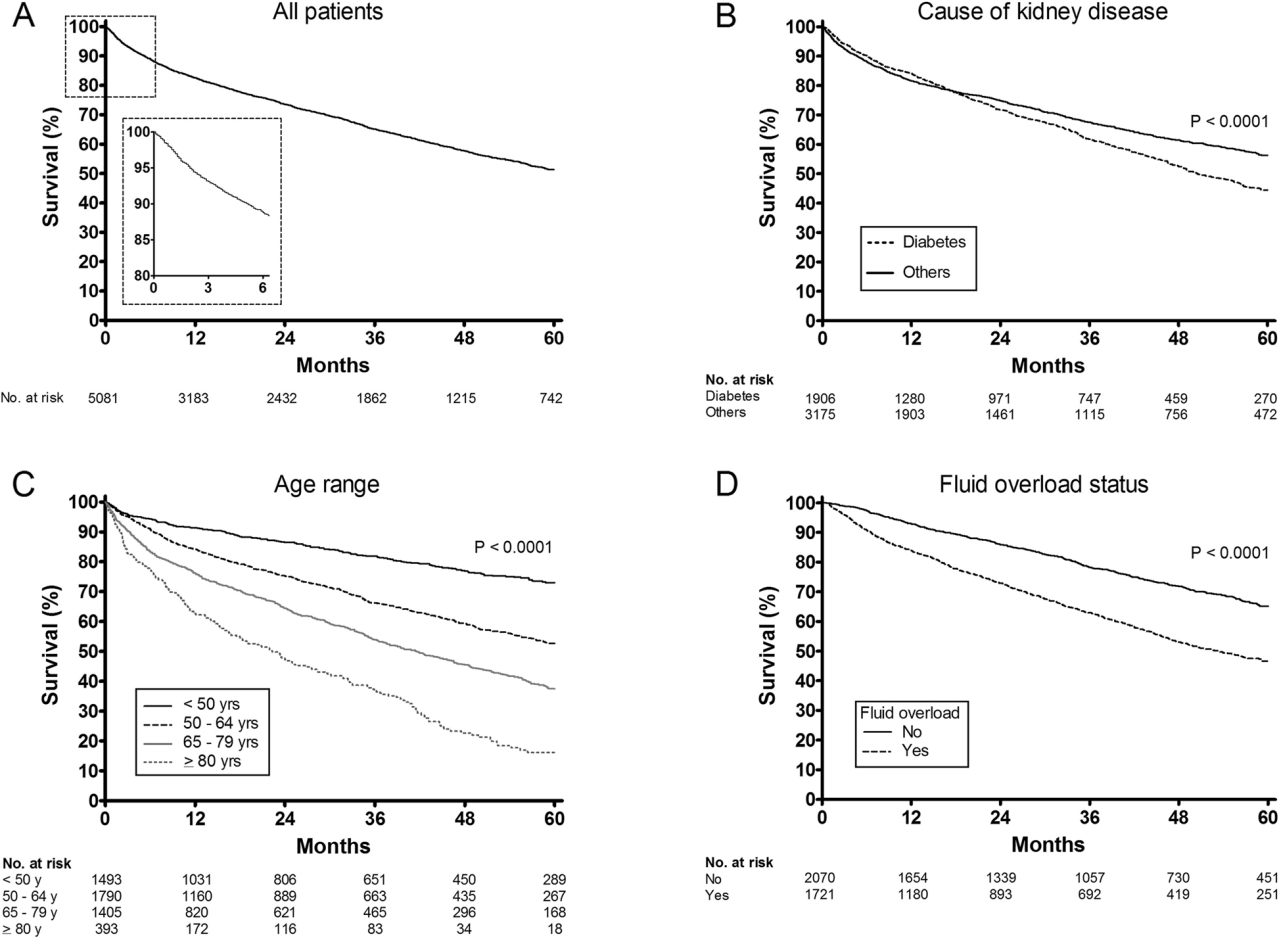
60-month Kaplan-Meier survival curves, with starting of follow-up on the admission to the dialysis centre. **A** - All incident patients; **B** - According to the cause of kidney failure (diabetes vs. other causes); **C** - According to the age range; and **D** - According to fluid overload status assessed by bioimpedance spectroscopy at haemodialysis entrance

The survival rates for all patients at 3, 12, 24, 36, 48 and 60 months of follow-up were 93.0, 82.6, 73.5, 65.1, 57.7 and 51.4%, respectively (Fig. 2A). Survival rate was lower for diabetics than non-diabetics (44.5% versus 56.3%; *p* < 0.0001), Fig. 2B; Also, it was significantly lower for the older patients: 73.0, 52.7, 37.5 and 16.2% for those < 50 years-old, 50 to 64 years-old, 65 to 80 years-old and ≥ 80 years-old, respectively (Fig. 2C). A total of 3.791 patients (74.6%) had BIS assessment at the admission, with 1.721 (45.4%) of them fluid overloaded. Survival rate was significantly lower among these patients, compared to those without fluid overload (46.6% versus 65.2%; *p* < 0.0001), Fig. 2D.

In the initial Cox regression model analysis with no adjustment, female gender, higher age, diabetes as cause of kidney failure, higher alkaline phosphatase, starting dialysis in the hospital, having catheter as initial vascular access, and fluid overload in BIS assessment were associated with higher death risk, whereas early referral to nephrologist, having higher body mass index (BMI), higher haemoglobin, transferrin saturation (TSAT), serum albumin, blood urea nitrogen (BUN), phosphorus, intact parathyroid hormone (iPTH), diastolic blood pressure and lean mass in BIS assessment were associated with reduction in the death risk. After adjustment, including in the model only variables with *p* values <0.10 in the univariate analysis, the following variables persisted associated with increased risk of death: age, diabetes, starting dialysis in the hospital, having catheter as initial vascular access, BMI, haemoglobin, TSAT, serum albumin, alkaline phosphatase, and fluid overload (Table 2).

**Table 2:** Cox regression for analysis of the risk of death in the 60-month period of follow-up, according to the baseline characteristics of the patients

	Unadjusted HR (95% CI)	***P***-value	Adjusted HR (95% CI)	***P***-value
Male	0.81 (0.74 – 0.90)	<0.0001	0.96 (0.83 – 1.11)	0.58
Age (years)	1.04 (1.03 – 1.04)	<0.0001	1.03 (1.03 – 1.04)	<0.0001
Non-white race/ethnicity	0.94 (0.85 – 1.04)	0.25	-	-
Diabetes	1.23 (1.12 – 1.36)	<0.0001	1.29 (1.11 – 1.50)	0.001
Public healthcare	1.01 (0.91 – 1.11)	0.87	-	-
Early referral	0.62 (0.55 – 0.70)	<0.0001	0.88 (0.74 – 1.05)	0.16
First dialysis in hospital	1.62 (1.43 – 1.83)	<0.0001	1.29 (1.05 – 1.58)	0.016
Catheter	2.01 (1.79 – 2.26)	<0.0001	1.22 (1.03 – 1.44)	0.023
BMI (Kg/m^2^)	0.97 (0.96 – 0.98)	<0.0001	0.98 (0.96 – 0.99)	0.015
Haemoglobin (g/dL)	0.87 (0.85 – 0.90)	<0.0001	0.95 (0.91 – 0.98)	0.004
TSAT ≥ 20%	0.67 (0.60 – 0.75)	<0.0001	0.80 (0.69 – 0.92)	0.002
Ferritin ≥ 200 ng/mL	1.09 (0.98 – 1.22)	0.12	-	-
Erythropoietin use	0.96 (0.87 – 1.05)	0.37	-	-
BUN (mg/dL)	0.99 (0.99 – 0.99)	<0.0001	1.00 (0.99 – 1.00)	0.13
Serum albumin (g/L)	0.92 (0.91 – 0.93)	<0.0001	0.96 (0.95 – 0.98)	<0.0001
Potassium (mEq/L)	0.98 (0.93 – 1.03)	0.48	-	-
Phosphorus (mg/dL)	0.89 (0.86 – 0.92)	<0.0001	1.00 (0.95 – 1.06)	0.92
Corrected calcium (mg/L)	1.02 (0.99 – 1.06)	0.14	-	-
iPTH (per 100 pg/mL)	0.95 (0.94 – 0.96)	<0.0001	1.00 (0.98 – 1.02)	0.81
ALP (per 100 UI/L)	1.09 (1.06 – 1.12)	<0.0001	1.09 (1.03 – 1.16)	0.005
Pre-HD SBP (mmHg)	1.00 (0.99 – 1.00)	0.34	-	-
Pre-HD DBP (mmHg)	0.98 (0.98 – 0.99)	<0.0001	1.00 (0.99 – 1.01)	0.89
Lean mass (%)	0.99 (0.98 – 0.99)	<0.0001	1.00 (0.99 – 1.00)	0.33
Fat mass (%)	1.00 (0.99 – 1.00)	0.51	-	-
Fluid overload	1.88 (1.66 – 2.14)	<0.0001	1.47 (1.27 – 1.71)	<0.0001

*HR* hazard ratio; *CI* confidence interval; *BMI* body mass index; *TSAT* transferrin saturation; *BUN* blood urea nitrogen; *iPTH* intact parathyroid hormone; *ALP* alkaline phosphatase; *HD* haemodialysis; *SBP* systolic blood pressure; *DBP* diastolic blood pressure

In the univariate analysis of subdistribution hazard ratio (sHR) by the Fine-Gray model, including kidney transplantation, migration to peritoneal dialysis and kidney function recovery as competing outcomes, the variables associated with death risk were the same found in the unadjusted Cox regression analysis. All those variables plus corrected calcium (*p* = 0.067) were included in the adjusted subdistribution proportional hazards model. Again, the variables associated with death risk were the same found in the Cox regression model, except having catheter as initial vascular access (*p* = 0.14), Table 3.

**Table 3:** Subdistribution hazard ratio (sHR) using the Fine-Gray method for analysis of the risk of death in the 60-month period of follow-up, according to the baseline characteristics of the patients

	Unadjusted sHR (95% CI)	***P***-value	Adjusted sHR (95% CI)	***P***-value
Male	0.83 (0.76 – 0.92)	0.0002	0.96 (0.82 – 1.11)	0.57
Age (years)	1.04 (1.03 – 1.04)	<0.0001	1.03 (1.03 – 1.04)	<0.0001
Non-white race/ethnicity	0.96 (0.87 – 1.06)	0.41	-	-
Diabetes	1.32 (1.20 – 1.45)	<0.0001	1.31 (1.12 – 1.53)	0.0006
Public healthcare	1.08 (0.98 – 1.19)	0.14	-	-
Early referral	0.72 (0.65 – 0.81)	<0.0001	0.88 (0.73 – 1.05)	0.15
First dialysis in hospital	1.58 (1.40 – 1.79)	<0.0001	1.31 (1.07 – 1.61)	0.009
Catheter	1.77 (1.58 – 1.99)	<0.0001	1.13 (0.96 – 1.34)	0.14
BMI (Kg/m^2^)	0.97 (0.96 – 0.98)	<0.0001	0.98 (0.96 – 0.99)	0.008
Haemoglobin (g/dL)	0.88 (0.86 – 0.91)	<0.0001	0.96 (0.92 – 0.99)	0.014
TSAT ≥ 20%	0.70 (0.63 – 0.78)	<0.0001	0.81 (0.70 – 0.94)	0.005
Ferritin ≥ 200 ng/mL	1.08 (0.96 – 1.20)	0.19	-	-
Erythropoietin use	0.97 (0.88 – 1.06)	0.49	-	-
BUN (mg/dL)	0.99 (0.99 – 1.00)	<0.0001	1.00 (0.99 – 1.00)	0.22
Serum albumin (g/L)	0.93 (0.92 – 0.94)	<0.0001	0.97 (0.95 – 0.98)	0.0002
Potassium (mEq/L)	1.01 (0.96 – 1.06)	0.69	-	-
Phosphorus (mg/dL)	0.90 (0.87 – 0.93)	<0.0001	1.00 (0.95 – 1.06)	0.99
Corrected calcium (mg/L)	1.03 (1.00 – 1.06)	0.067	1.01 (0.96 – 1.06)	0.67
iPTH (per 100 pg/mL)	0.96 (0.95 – 0.98)	<0.0001	1.00 (0.98 – 1.02)	0.89
ALP (per 100 UI/L)	1.09 (1.06 – 1.12)	<0.0001	1.08 (1.02 – 1.14)	0.007
Pre-HD SBP (mmHg)	1.00 (1.00 – 1.00)	0.67	-	-
Pre-HD DBP (mmHg)	0.98 (0.98 – 0.99)	<0.0001	1.00 (0.99 – 1.01)	0.96
Lean mass (%)	0.99 (0.98 – 0.99)	<0.0001	1.00 (0.99 – 1.00)	0.17
Fat mass (%)	1.00 (1.00 – 1.01)	0.26	-	-
Fluid overload	1.93 (1.72 – 2.17)	<0.0001	1.50 (1.29 – 1.74)	<0.0001

*CI* confidence interval; *BMI* body mass index; *TSAT* transferrin saturation; *BUN* blood urea nitrogen; *iPTH* intact parathyroid hormone; *ALP* alkaline phosphatase; *HD* haemodialysis; *SBP* systolic blood pressure; *DBP* diastolic blood pressure

## Discussion

This observational study involving more than 5,000 patients contributes to outlining the profile of the incident patients on HD in an emerging country, which has one of the largest dialysis population in the world and sheds light on the main risk factors associated with their long-term risk of death. This information can be particularly valuable for the local health policy of developing countries since the vast majority of available data is derived from developed nations.

The 5-year survival rate in our study was higher than that usually seen in developed countries, excepting Japan [4]. According to the ERA Registry Annual Report 2019 [3], the unadjusted 5-year survival rate of dialysis incident patients in the period 2010-2014 in Europe was 42.3%. Such difference could be explained by the markedly lower age and a much lower rate of kidney transplantation in our population, keeping younger and healthier patients on dialysis, consequently improving overall survival in the modality. As our incident dialysis population grows progressively older, an increase in mortality can be expected.

Indeed, we can see that the profile of the population incident on HD in Brazil is progressively catching up the ones of the developed countries accompanied by a trend of a negative impact in the survival rate. When comparing the population of the present study with the incident patients on HD between 2000 and 2004 in the same facilities chain in Brazil [10], the age at admission increased from 52 to 59 years, and diabetes as the underlying kidney disease rose from 20.4% to 37.5%. Accordingly, the 5-year survival rate decreased from 58.2% to 51.4%. This could be justified not only by the differences of age and prevalence of diabetes between the two periods, but also by the inclusion of early deaths. In the present study, the mortality rate was 7% in the first 90 days on dialysis, which was proportionally much higher than throughout the remaining follow-up period, whereas in the previous study, patients who died in less than 90 days were excluded.

As expected, we found a strong association between both, older age and diabetes as the underlying kidney disease, with the risk of death. Likewise, we observed a significant increase in the risk of death for patients who started unplanned dialysis at a hospital. Unfortunately, most patients started under such circumstances, despite the wide recognition of that condition as a potentially modifiable risk factor [11], bringing to light the shortcomings of CKD management in the pre-dialysis phase by our health care system, either public or private.

Regarding laboratory variables on admission, higher serum albumin levels were associated with a lower death risk, which is a well-known association [12]. However, we cannot imply whether this would be a modifiable risk factor or just a marker of underlying clinical conditions not addressed in the analysis.

Higher alkaline phosphatase levels, but not iPTH, was associated with an increased risk of death. It is possible that serum alkaline phosphatase levels better reflect the bone mineral disorder, whereas it is known that iPTH levels in haemodialysis patients, in a wide range, varying from 2 to 9 times the upper limit of normality in the general population, have a weak correlation with histopathological findings on bone biopsy [13]. Similarly, a previous study showed the association between serum alkaline phosphatase levels and mortality risk in patients with advanced CKD transitioning to dialysis. Higher alkaline phosphatase levels over the last 6 months before initiation of dialysis were independently associated with increased post kidney failure mortality risk [14].

Almost half of the patients had no anaemia on admission and the vast majority did not present low haemoglobin levels that would demand blood transfusions. Most patients starting dialysis at a hospital may have had their anaemia treated and compensated for before discharge. In addition, outpatients were already undergoing care by nephrologists. Consistent with such interpretation, 44% of patients were already on EPO prescription at admission. We found an association between high haemoglobin levels at admission and low risk of death. It is interesting because the anaemia status is changed soon after HD start, with adequate EPO and iron replacement, targeting the same haemoglobin range for all patients along the follow-up period. The lower risk of death for patients with higher haemoglobin levels may reflect a better clinical condition not fully detected by the adjusted analysis model in the study or the need for higher EPO doses over the follow-up period in the patients who initially had lower haemoglobin levels.

It is interesting the association between TSAT ≥ 20% and the lower risk of death seen in the present study. Such association was found and well explored in a recent CKDopps study with pre-dialysis chronic kidney disease (CKD) patients [15]. However, whether low TSAT was just a marker of inflammation/malnutrition [16, 17] or iron replacement would be effective to improve outcomes is a question that could only be answered through interventional studies. According to the PIVOTAL study [18], a more vigorous iron replacement in patients on maintenance haemodialysis, but deliberately avoiding ferritin levels ≥ 700 ng/mL or TSAT ≥ 40%, improved anaemia control, reduced erythropoiesis-stimulating agent doses and significantly reduced the risk of unfavourable clinical outcomes as compared to the control group, randomized to receive iron reactively when ferritin < 200 ng/mL or TSAT < 20%. Thus, based on the findings of that study, we can assume that intravenous iron replacement for our incident patients with TAST < 20% could have a favourable effect on outcomes.

Patients with a higher BMI were shown to have a lower risk of death, confirming a well-known association seen in previous studies. This would happen because in the haemodialysis population, with an unacceptably high mortality rate, the benefits of the obesity-related nutritional reserve would probably outweigh the cardiovascular risks associated with the metabolic disorders, having such phenomenon been called the “obesity paradox” [19]. Interestingly, in the present study, neither the fat mass index nor the lean mass index measured by BIS were significantly associated with death risk.

The presence of FO in a single assessment on admission was the variable that was found to have the strongest association with the risk of death, even in such a long follow-up period. However, it is questionable whether FO would be directly associated with the cause of death or would be just a marker of other clinical conditions with poor prognosis, such as heart failure, lower residual urinary volume or hypoalbuminemia, contributing to the extracellular volume expansion. Even if such clinical conditions are behind FO, the way the patients are treated forward and how FO is corrected will probably impact the outcomes. Indeed, the long-term exposition to excessive extracellular volume is a much stronger predictor of death risk than the presence of FO at admission on HD, as shown by Zoccali et al [8]. We demonstrated in a previous study that even prevalent patients with FO while on conventional HD, after switching to short daily HD and subsequent correction of FO, had the same survival rate as those patients on short daily HD who were not with FO when they were still on conventional HD [20]. This finding corroborates once again that FO would be a modifiable risk factor and that its correction should be considered a priority in the clinical management.

Our study has several limitations. The first is the retrospective nature of the analysis. Second, the lack of BIS assessment on about a quarter of patients. This was because the random lack of registration of BIS evaluation in the electronic database in some clinics, especially in the first years, and also due to a significant number of patients who indeed did not undergo BIS assessment, especially those who were readmitted or died early. Another limitation would be some lack of representativeness of the patients in this study compared to the haemodialysis population in Brazil as a whole. Almost half of the patients in this study were from facilities located in the state of Rio de Janeiro, where only about 10% of the Brazilian population lives, and 42% of them had their treatment funded by the private healthcare system, while the average in Brazil is about 20% [1]. Anyway, the characteristics of patients funded by the public and private systems are presented separately in the supplementary material (Table S1). This study also has its strengths, such as the large number of patients, the long period of follow-up and the inclusion of variables, such as BIS assessment.

## Conclusion

This study gives an overview of a large CKD population incident on maintenance haemodialysis in a developing country and provides information on factors, several of them potentially modifiable, such as unplanned initiation of dialysis in the hospital and fluid overload, which were found to be associated with the risk of death over a 5-year period.

## Supplementary Information

**Additional file 1.**

## Data Availability

The datasets used and analysed during the current study are available from the corresponding author on reasonable request.

## Abbreviations

BIS: Bioimpedance spectroscopy
CVC: Central venous catheter
ERA-EDTA: European Renal Association – European Dialysis and Transplant Association
FO: Fluid overload
HD: Haemodialysis
USRDS: United States Renal Data System
