# Supplementary material for: Characteristics and predictors of mortality on haemodialysis in Brazil: a cohort of 5,081 incident patients
Source: BMC Nephrol. 2022 Feb 23;23:77. doi: 10.1186/s12882-022-02705-x (PMC8864821; doi:10.1186/s12882-022-02705-x)
Supplement: Supplementary file 1 — Additional file 1. [file 12882_2022_2705_MOESM1_ESM.docx]

| **Table S1.** Baseline characteristics of patients according to the source of payment. | | | |
| --- | --- | --- | --- |
| Characteristic | Public  (n= 2,951) | Private  (n= 2,130) | *P*-value |
| Gender male, n (%) | 1,713 (58.0) | 1,305 (61.3) | 0.023 |
| Age, years | 58 (46 – 67) | 61 (49 – 72) | <0.0001 |
| Non-white race/ethnicity, n (%) | 1,877 (63.6) | 1,000 (46.9) | <0.0001 |
| Primary cause of kidney failure, n (%) |  |  |  |
| Diabetes | 1,040 (35.8) | 866 (40.6) | <0.0001 |
| Hypertension | 837 (28.4) | 480 (22.5) | <0.0001 |
| Glomerulonephritis | 337 (11.4) | 210 (9.9) | <0.0001 |
| Polycystic kidney disease | 97 (3.3) | 96 (4.5) | 0.032 |
| Others | 182 (6.2) | 175 (8.2) | 0.006 |
| Unknown | 459 (15.6) | 302 (14.2) | 0.19 |
| Early referral to nephrologist, n (%) | 1,057 (35.8) | 925 (43.4) | <0.0001 |
| Place of first dialysis session |  |  |  |
| Hospital | 1,707 (57.9) | 1,353 (63.5) | <0.0001 |
| Dialysis unit | 817 (27.7) | 504 (23.7) | 0.001 |
| No information | 427 (14.5) | 273 (12.8) | 0.098 |
| Initial vascular access, n (%) |  |  |  |
| Native arteriovenous fistula | 933 (31.6) | 576 (27.0) | <0.0001 |
| Graft | 21 (0.7) | 14 (0.7) | 0.95 |
| Temporary catheter | 1,815 (61.5) | 1,032 (48.5) | <0.0001 |
| Tunnelled catheter | 182 (6.2) | 508 (23.8) | <0.0001 |
| Hepatitis B infection, n (%) | 24 (0.8) | 9 (0.4) | 0.13 |
| Hepatitis C infection, n (%) | 87 (2.9) | 49 (2.3) | 0.19 |
| HIV infection, n (%) | 24 (0.8) | 24 (1.1) | 0.32 |
| Erythropoietin use, n (%) | 1,278 (43.3) | 953 (44.7) | 0.32 |
| Haemoglobin, g/dL | 9.8 (8.2 – 11.4) | 9.8 (8.5 – 11.5) | 0.99 |
| Transferrin saturation, % | 25 (17 – 36) | 23 (16 – 32) | <0.0001 |
| Ferritin, ng/mL | 390 (170 – 773) | 320 (134 – 650) | <0.0001 |
| BUN, mg/dL | 55 (43 – 70) | 54 (42 – 67) | 0.018 |
| Serum albumin, g/L | 36 (32 – 39) | 37 (33 – 40) | <0.0001 |
| Potassium, mEq/L | 5.1 (4.5 – 5.8) | 5.0 (4.4 – 5.7) | 0.013 |
| Phosphorus, mg/dL | 4.6 (3.7 – 5.7) | 4.5 (3.6 – 5.6) | 0.007 |
| Corrected calcium, mg/L | 9.0 (8.5 – 9.5) | 9.1 (8.6 – 9.5) | 0.74 |
| Intact parathyroid hormone, pg/mL | 305 (150 – 575) | 211 (97 – 411) | <0.0001 |
| Alkaline phosphatase, UI/L | 99 (75 – 143) | 92 (70 – 126) | 0.0001 |
| Pre-HD Systolic BP, mmHg | 143 (130 – 157) | 140 (128 – 155) | <0.0001 |
| Pre-HD Diastolic BP, mmHg | 80 (73 – 87) | 76 (68 – 83) | <0.0001 |
| Body mass index, Kg/m^2^ | 23.4 (20.8 – 26.4) | 24.2 (21.4 – 27.6) | <0.0001 |
| Bioimpedance spectroscopy assessment |  |  |  |
| Lean mass, % | 51.0 (41.1 – 62.3) | 47.4 (38.0 – 57.5) | <0.0001 |
| Fat mass, % | 32.9 (24.3 – 40.2) | 35.6 (27.7 – 42.9) | <0.0001 |
| Excessive extracellular volume, % | 12.9 (4.7 – 21.1) | 12.8 (4.9 – 21.1) | 0.99 |
| Fluid overload, n (%) | 996 (44.8) | 725 (46.2) | 0.85 |
| Values are expressed as frequency (%) or median (interquartile range). BUN, blood urea nitrogen; HD, haemodialysis; BP, blood pressure. | | | |
